# Selected Abstracts from the Meeting of “Molecular Mechanisms of Autophagy in Diseases”

**DOI:** 10.1038/s41420-021-00587-w

**Published:** 2021-09-01

**Saint-Petersburg, October 30-31, 2020**

**Sponsorship:** Publication of this supplement was funded in part by the Russian Government Program for the Recruitment of the leading scientists into the Russian Institutions of Higher Education grant# 14.W03.31.0029, and Monomax, LLP, which has organized the meeting, collected and prepared the abstracts for publication.

All content was reviewed and approved by the Scientific Committee at the Institute of Cytology, which held full responsibility for the abstract selections.

Webpage for the event: http://cancerproteostasis.com

## 1

### The role of autophagy in chemotherapy resistance of cisplatin in ovarian cancer

#### Lamak Alsoulaiman^1^, Elnara Biktagirova^2^, Zinaida Abramova^2^

##### ^*1*^*Moscow Institute of Physics and Technology, Moscow, Russia;*^*2*^*Kazan Federal University, Kazan, Russia*; ORCID: 0000-0003-3749-3411

**Introduction:** Epithelial ovarian cancer is one of the most lethal oncogynecological diseases. Recent researches showed that autophagy induction contributes to the development of the resistance against anticancer drugs. In this work, we evaluated the contribution of the autophagy to cisplatin resistance in the ovarian cancer cell line OVCR8. Atg5 protein plays a critical role in the initiation and elongation stages of autophagy. Effective knockout for ATG5 blocks autophagy induction in the ovarian cancer cell lines, which is confirmed by the lower expression level of the LC-II.

**Materials and methods:** In this study, we knocked out *ATG-5* in the ovarian cell cancer OVCAR8 using doxycycline-inducible CRISPR-Cas9.

**Results:** After 6 days in the doxycycline containing media, the Atg-5 protein was effectively depleted. The western blot showed the absence of LC3-II (a marker of autophagy) in comparison with wild-type, which means that we successfully inhibited autophagy. We defined the cisplatin IC50 for *ATG5-*knockouted OVCAR8 (IC50= 2.14 mkM) and wild-type cell line (IC50=2.74 mkM). Fold Change (FC) = IC50^mut^: IC50^w^ = 0.78. We concluded that cells with inhibited autophagy are more sensitive to cisplatin than the wild type. Our data is similar to studies suggesting that autophagy can defend ovarian cancer-associated fibroblasts from oxidative stress. That means blocking autophagy can sensitize ovarian cancer-associated fibroblasts for chemotherapeutic drug cisplatin.

We evaluated the expression levels of autophagy markers (LC3-I, LC3-II, P62, Atg5), and anti-apoptotic protein (Bcl2).

The western blot showed non expression of LC3-II (a marker of autophagy) in comparison with wild-type. However other autophagy markers P62 and LC3-I were downregulated in Atg5-knockouted cell. The p62 depletion contradicts the published literature.

**Conclusion:** We are hypothesizing that in ovarian cancer cells, p62 level is regulated by uncharacterized signaling cascade that senses level of the autophagy. The exact mechanism of this process in autophagy- missing cells is not clear. In addition, in Atg5-knockouted cells, Bcl2 levels were higher than wild-type (p-value < 0.05*) and high level of Bcl2 inhibits apoptosis, and contributes to the resistance to chemotherapeutic drugs, which is consistent with earlier published Results. Autophagy is a promising therapeutic target and characterization of autophagy-mediated chemotherapeutic resistance will pave the way to better treatment of the ovarian cancers.

**Disclosure:** The authors confirm that they have no conflict of interest.

## 2

### May polyunsaturated fatty acid oxidation be linked to excessive mitophagy and metatastatic progression in hepatocellular carcinoma?

#### Plamena Angelova^1^, Nikolay Pestov^2^, Mikhail Shchepinov^3^, Andrey Abramov^1,4^

##### ^1^UCL Queen Square Institute of Neurology, Queen Square, London, UK; ^2^Moscow Institute of Physics and Technology, Moscow, Russia; ^3^Retrotope Inc., Los Altos, USA; ^4^Orel State University, Orel, Russia; ORCID: 0000-0002-9973-0120

**Introduction:** A significant number of studies favors the view that excessive autophagy is an important hallmark of transition from benign to aggressive metastatic tumors. In hepatocellular carcinoma, this is partially mediated by p53 phosphorylation at S-392. Mitophay is an evolutionary conserved organelle-selective component of autophagy that is targeted at dysfunctional mitochondria through their degradation in the autophagosome. Mitophagy plays a critical role in mitochondrial integrity and is frequently impaired in some cancers. Both iron chelation and iron supplementation promote mitophagy demonstrating that normal mitochondrial balance between fusion and fission, between formation of new mitochnodria and death of old organelles is sensitive to various stresses. Fission promotes recovery from oxidative stress and increases metastasis.

**Results:** Palmitic acid as well other free fatty acids are known to increase mitochondrial oxidative stress and initiation of mitophagy. Effect of PUFAs is complex: ω6-PUFA induces autophagy but reduces antioxidant ability, whereas ω3-PUFAs decrease autophagy and, specifically, mitophagy. We have obtained preliminary data that cells exhibit characteristics of reduced mitochondrial health when compared to those age-matched control subjects. These include changes in mitochondrial morphology, number, and function. Incubation of these cells with D2-PUFAs for 72 hours restores these parameters to control levels. The structural and functional changes in mitochondria derived from these subjects were associated with increased rates of LPO, and coincident reductions in glutathione levels. These changes were also reversed with D2-PUFA treatment.

**Conclusions:** We hypothesize that the excessive oxidation of cellular lipids, especially PUFAs, exacerbate mitochondrial abnormalities. Yet, a direct link between oxidation of PUFAs and regulation of mitophagy in cancer has to be established.

**Disclosure:** The authors confirm that they have no conflict of interest.

## 3

### Establishment of cell line xenografts and PDX models for studying autophagy and apoptosis in response to chemotherapy in AML

#### Ekaterina Baidyuk^1^, Marina Vasyutina^2^, Larisa Girshova^2^, Alexey Petukhov^1,2^, Andrey Zaritsky^2^, Ekaterina Belotserkovskaya^2^, Oleg Demidov^1,3^

##### ^1^Institute of Cytology of the Russian Academy of Sciences, Saint-Petersburg, Russia; ^2^Almazov National Medical Research Center, Saint-Petersburg, Russia; ^3^INSERM U866, University of Burgundy, Dijon, France; ORCID: 0000-0002-4108-9932

**Introduction:** Recently, the patient-derived xenograft (PDX) models in mice have significantly improved the process of preserving and amplification of tumor tissues for biomedical research and the optimization of personalized therapeutic protocols. Acute myeloid leukemia (AML) is a frequent hematological malignancy characterized by high lethality and frequent recurrence. Despite the recent addition of targeted therapy and the use of epigenetic drugs, the main drug of choice for AML treatment remains cytarabine. As was shown before, cytarabine can induce autophagy that seriously compromises the efficiency of leukemic cell elimination. The AML cells are hard to cultivate in vitro and their transplantation into immunodeficient mice has a low rate of graft success. A new model of NOD SCID gamma immunodeficient mice (NSG) bearing transgenic insertion of human cytokines (NSG-SGM3) was developed to improve the grafting rate of human myeloid cells. Here we describe an AML PDX model NSG-SGM3 in comparison with xenografts of established human AML cell lines that can be used for the more clinically relevant analysis of autophagy and other cell death types.

**Materials and methods:** The bone marrow mononuclear fraction of cells from four AML patients undergoing treatment in Almazov’s Medical Center were isolated by gradient centrifugation. The cells from each patient were injected into the tail vein of NSG-SGM3 mice. Similarly, OCI-AML2 (M4) and HL60 (M2) AML cell lines were transplanted into NSG-SGM3 mice. The success of grafting was monitored by blood formula count, PCR, and FACS analysis with anti-human CD45 antibodies.

**Results:** As expected, all transplantations with AML cell lines were successfully grafted in NSG-SGM3

mice in comparison with the much lower rate of graft success with primary AML PDXs. The successful grafting in mice was confirmed by more than 10 times increased number of leucocytes in the blood, positive PCR reaction to the human-specific region of PPM1D gene, and the presence of human-CD45 positive cells in the bloodstream. The blast forms of myeloid cells were observed in the blood of mice transplanted with primary AML PDX. The primary AML PDX mice developed first symptoms in 3 weeks and start dying in 6 weeks after transplantation. Mice transplanted with AML cell lines start dying earlier, in 4 weeks after transplantation. These mice showed an appearance of multiple neoplasms throughout the body (subcutaneous, intramuscular, on internal organs). In contrast to mice grafted nearly AML PDX, all mice grafted with AML cell lines developed the symptoms of neuroleukemiosis that is a frequent complication of AML in human patients. The neuropathic symptoms were manifested both in mice behavior: apathy, slow movement, gait unsteadiness, impaired coordination, and in noticeable physical disorders, such as paralysis of the hind limbs (AML2), enlargement and asymmetry of the skull, enophthalmos, inflammation of the brain membranes (HL60).

**Conclusions:** We concluded that previously established AML cell lines OCI-AML2 and HL60 have a high rate of successful grafting and developed a more aggressive AML-like state in NSG-SGM3 immunodeficient mice than primary AML PDX. The neuroleukemiosis can be successfully modeled by AML cell lines, but not by primary AML PDX. The higher tumorigenicity of previously cultivated in vitro AML cells may lead to atypical behavior in response to therapy. The primary AML PDX without previous cultivation in vitro could be a better, more relevant model for developing a personalized treatment for AML patients and studying cell death autophagy in leukemic cells.

**Funding:** Russian Science Foundation Grant 19-75-20128.

**Disclosure:** The authors confirm that they have no conflict of interest. The work was supported by the Russian Science Foundation grant 19-75-20128.

## 4

### Recombinant analog of human protein SLURP-1 inhibits activity of autophagy-related intracellular signaling pathways in skin adenocarcinoma cells

#### Maxim Bychkov^1^, Olga Shlepova^1,2^, Zakhar Shenkarev^1,2^, Ekaterina Lyukmanova^1,2,3^, Mikhail Shulepko^1^

##### ^1^Shemyakin-Ovchinnikov Institute of Bioorganic Chemistry of the Russian Academy of Sciences, Moscow, Russia; ^2^Moscow Institute of Physics and Technology (State University), Moscow, Russia; ^3^Lomonosov Moscow State University, Moscow, Russia; ORCID: 0000-0001-8504-5256

**Introduction:** Autophagy is the degradation of unnecessary or dysfunctional cellular components, that is regulated by multiple intracellular signaling pathways. Dysregulation of autophagy promotes survival of the malignant cells and supports tumor progression. Thus, inhibition of autophagy can be a useful strategy for cancer treatment. Recently, we have shown that the recombinant analog of human epithelial homeostasis regulator SLURP-1 (rSLURP-1) effectively suppresses proliferation of epithelial cancer cells by modulating many intracellular signaling pathways. The goal of the current study was an investigation of the rSLURP-1 influence on intracellular pathways, implicated in autophagy control.

**Materials and methods:** The analysis of the phosphorylation of intracellular kinases and transcription factors, implicated in autophagy in A431 skin carcinoma cells revealed that upon 1 h incubation rSLURP-1 suppresses the activity of multiple intracellular pathways that mediate autophagy. Particularly, rSLURP-1 inhibited ERK-RSK autophagic axis, suppressed phosphorylation of JNK that drives LC3 cleavage, inhibited AKT, p70 s6 kinase, p38α MAP kinase, and Src family kinases (Src and Lyn) activity, which are all capable to activate autophagy in cancer cells. Moreover, rSLURP-1 increased phosphorylation of mTOR activator PRAS40 and WNK-1, which both are known to inhibit autophagy, and inhibited expression of β-catenin, which is usually up-regulated during autophagy. Incubation of A431 cells with rSLURP-1 during 1 h led to activation of mTOR inhibitor GSK3α/β, that generally activates autophagy. We propose that active GSK3α/β cooperates with mTOR activator PRAS40 or p70 s6 kinase for the regulation of AKT/mTOR signaling pathway, and altogether these regulators inhibit autophagy by inhibition of AKT and restriction of mTOR activation. Moreover, rSLURP-1 simultaneously activated expression of HSP60, which is known to inhibit autophagic flux in cancer cells and decreased phosphorylation of HSP27, which usually stimulates autophagy. rSLURP-1 action on autophagy-related proteins was not limited by action on kinases, but also included transcriptional control of autophagy. rSLURP-1 inhibited phosphorylation of pro-autophagic transcriptional factors, such as nuclear STAT3, CREB, STAT6, and Chk-2 kinase and simultaneously activated STAT5, which is known to repress autophagy in cancer cells. rSLURP-1 also activated p53 tumor suppressor protein, which can be implicated in autophagy control by inhibition of Src kinase.

**Results:** All inhibitory effects of rSLURP-1 on autophagy-related molecules were evanesced upon 24 h incubation of A431 cells with rSLURP-1, that points on A431 cells adaptation to rSLURP-1 action on autophagy-related protein activity. Moreover, after 24 h incubation with rSLURP-1 A431 cells showed significant activation of ERK1/2 kinases, elevated expression of β-catenin, and enhanced activity of CREB and Chk-2. Western blot analysis did not reveal the cleavage of LC3 autophagy marker in A431 cells upon 24 h incubation with rSLURP-1, and cell viability assays did not show a deterioration of rSLURP-1 antiproliferative activity on A431 cells upon 48 h incubation. Nevertheless, inhibition of autophagy can become a perspective strategy for overcoming of A431 cells resistance to rSLURP-1 upon prolonged incubations.

**Conclusions:** Thus, for the first time we showed that rSLURP-1 simultaneously inhibits autophagy activators and up-regulates anti-autophagic protein activity in A431 carcinoma cells upon 1 h incubation. We also suppose that inhibition of autophagy can be a perspective strategy for overcoming carcinoma cell resistance to rSLURP-1 upon prolonged incubations.

**Funding:** The study was supported by Russian Science Foundation (project # 17-74-20161).

**Disclosure:** The authors confirm that they have no conflict of interest. The study was supported by Russian Science Foundation (project # 17-74-20161).

## 5

### Set7/9 controls the balance between apoptosis and autophagy

#### Alexandra Daks^1^, Oleg Shuvalov^1^, Matvey Ivanov^1^, Oleg Semenov^1^, Olga Fedorova^1^, Sergey Parfenyev^1^, Arsenia Zharova^1^, Nickolai Barlev^1^

##### ^1^Institute of Cytology of Russian Academy of Science, St. Petersburg, Russia; ORCID: 0000-0003-0495-1244

**Introduction:** Recent studies suggest that autophagy plays an adaptive role in tumor cells exposed to anticancer treatment. Currently, the autophagy inhibitor hydroxychloroquine (HCQ) is in clinical trials as a part of combined anticancer therapy for different types of tumors. However, HCQ is known to have severe side effects including retinopathy. Therefore, the search for new inhibitors of autophagy is highly relevant.

Lysine-specific methyltransferase Set7/9 methylates both histones (H1 and H3) and non-histone targets and plays important roles in proliferation, cell cycle regulation, EMT, DNA damage repair, and apoptosis. To date the role of Set7/9 in autophagy is poorly understood. Thus, we investigated the effect of Set7/9 methyltransferase on autophagy and apoptosis in several human cancer cell lines under genotoxic stress conditions.

**Materials and methods:** GST-pulldown assay coupled with LC-MS/MS mass-spectrometry was used to identify the Set7/9 interacting proteins. Several human breast and lung cancer cell lines (H1299, A549, MCF7, and SKBR3) with different status of Set7/9 were generated using the CRISPR/Cas9 editing system. Cytotoxicity after doxorubicin treatment was measured by MTT test. The autophagy levels were tested using Western blotting analysis of LC3 and p62 proteins, as well flow cytometry after lysotracker staining. Analysis of apoptosis level was performed by flow cytometry after Annexin V-PI staining.

**Results:** Analysis of GST-pulldown assay coupled with mass-spectrometry has identified, among several interacting proteins, a novel Set7/9-interacting partner, HMBG1. This protein is known to form the complex with Beclin-1 and promote autophagy.

Using the panel of cancer cell lines, we showed that depletion of Set7/9 diminished the level of autophagy in these cells after Chloroquine treatment. Importantly, this effect was reproduced when specific small-molecular inhibitor of Set7/9, (R)-PFI-2, was used on cells with normal level of Set7/9. Since autophagy protects cancer cells from death induced by genotoxic drugs, we tested whether the (R)-PFI-2 can synergize with doxorubicin, an inhibitor of Topoisomerase II. Indeed, we found that the depletion or inhibiting of Set7/9 sensitized human breast and lung cancer cells to doxorubicin, etoposide and cisplatin and caused apoptosis.

**Conclusions:** We showed that the suppression of Set7/9 shifts the balance between autophagy and apoptosis towards apoptosis via the attenuation of autophagy. Thus, the pharmacological inhibition of Set7/9 may be considered as a novel anti-cancer therapeutic approach.

**Funding:** This work was supported by Russian Science Foundation Project No 19-75-10059.

**Disclosure:** The authors confirm that they have no conflict of interest. This work was supported by Russian Science Foundation Project No # 19-75-10059.

## 6

### TG2-induced autophagy in response to DNA damage

#### Olga Fedorova^1^, Sergey Parfenyev^1^

##### ^1^Institute of Cytology of the Russian Academy of Sciences, St.Petersburg, Russia; ORCID: 0000-0002-0531-4389

**Introduction:** Transglutaminase 2 (TG2) is multifunctional enzyme which catalyze protein modifications (crosslinking reaction). TG2 displays transamidation reaction, it acts ATPase, GTPase, protein kinase, and protein disulfide isomerase. Depending on stimuli, TG2 can change biological activities and may exert anti- and pro-apoptotic effects. TG2 transamidation activity is an important role in the assembly of protein aggregates and autophagosome maturation. TG2 dysfunction associated with different diseases including cancer, fibrosis, inflammation, neurodegenerative and cardiovascular diseases.

The aim of our study was to determine the role of TG2 in cell response to DNA damage.

**Materials and methods:** We used wild-type (WT MEF) and TG2 knockout (KO MEF) MEF cell lines in this study. To assess survival cells MTT test and LDH cytotoxicity assay were used. To detect apoptosis Annexin V-FITC assay was performed. The level of autophagy was tested by Lysotraker staining and assessment the level of expression of LC3 and p62.

**Results:** Using different cell viability assays we show that decreased expression of TG2 leads to sensitization cells to DNA damage agents (doxorubicin and etoposide). Using annexin V assay we have shown that suppression of TG2 promotes apoptosis in response to etoposide. It is known that TG2 promotes autophagy by induction LC3. We study the influence of DNA-damage agents (doxorubicin and etoposide) on induction of autophagy in TG2^-/-^ and control cell lines. We have shown that suppression of TG2 inhibits autophagy and activates apoptosis after treatment of DNA damage agents.

**Conclusions:** We assume that inhibition autophagy by suppression of TG2 promotes activation apoptosis and increase sensitivity to DNA damage agents.

**Funding:** This work was supported by RSF research project 18-75-10076.

**Disclosure:** The authors confirm that the project was partly supported by the RSF research project 18-75-10076.

## 7

### Interaction of TG2 with p73 upon autophagy

#### Yuliya Gnennaya^1^, Mauro Piacentini^2^

##### ^1^Institute of Cytology of the Russian Academy of Sciences, St. Petersburg, Russia; ^2^University of Rome “Tor Vergata”, Rome, Italy; ORCID: 0000-0002-3571-333X

**Introduction:** Transglutaminase 2 (TG2) is a conservative Ca2+ -dependent multidomain enzyme belonging to the family of transglutaminases, which has a number of enzymatic functions, such as: transamidase, serine-threonine kinase, disulfide isomerase, protein kinase, and GTP-ase. In recent years, it has been established that TG2 plays a key role in regulating the balance of such important processes as apoptosis and autophagy, regulating the survival of cancer cells, and, as a result, can be an innovative biomarker and therapeutic target in various types of cancer.

**Materials and methods:** The transcription factor p53 is considered as one of the major tumor suppressors. According to the literature, TG2 can promote aggregation and depletion of p53 through autophagy in renal carcinoma cells, while suppression of endogenous TG2 in neuroblastoma cells leads to a significant increase in the activity of p53, including its phosphorylated forms. Recently, it was found that TG2 promotes autophagy and correlates with the expression of LC3, which marks the formation of autophagosomes. At the same time, on the nature and extent of inducing stress, p53 is able to variously modulate the autophagy process.

**Results:** p53 has two homologs, p63 and p73, which together form the p53 family. Expression of the transactivation-competent isoform Tap73γ correlates with the accumulation of LC3II and DRAM through genotoxic stress; however, unlike p53-mediated autophagy, DRAM was not involved in the formation of autophagosomes. However, the detailed mechanism of signal transmission of p73 for the induction of autophagy is unknown. The question also remains open whether p73 can lead to degradation of LC3 under the cell starvation conditions and whether TG2 participates in this process.

**Conclusions:** Thus, the goal of the future work is to demonstrate the involvement of p73 in the context of TG2-mediated autophagy.

**Funding:** The research is supported by: Mega-Grant Program 14.W03.31.0029 and the Russian Science Foundation Project No 21-75-10138.

**Disclosure:** The authors confirm that the project was supported by ﻿Mega-Grant Program 14.W03.31.0029 and the Russian Science Foundation Project No 21-75-10138.

## 8

### Wip1 reduces the resistance of AML cells to chemotherapeutic drugs

#### Vasily Golotin^1^, Oleg Demidov^1,2^

##### ^1^Institute of Cytology of the Russian Academy of Sciences, Saint-Petersburg, Russia; ^2^INSERM, University of Burgundy, Dijon, France; ORCID: 0000-0003-0385-2463

**Introduction:** Acute myeloblast leukemia (AML) is a frequent hematological malignancy that arises spontaneously or can be induced by chemotherapy or ionizing radiation. The first-line treatment for AML patients includes chemotherapeutic drugs such as cytarabine and etoposide. Both drugs can induce autophagy in AML cells that explains the frequent resistance of leukemic cells to therapy (Bosnjaket al., 2014). Wild-type p53 induced phosphatase (Wip1) is a potential regulator of autophagy. Wip1 can modify phosphorylation and activity of several autophagy-controlling proteins such as Ulk1 and mTOR (Torii et al., 2016). Here we investigated the effect of Wip1 inhibition on the sensitivity of AML cells to cytarabine treatment.

**Materials and methods:** The initial sensitivity of cells to cytarabine (a.k.a. Cytozar) was tested by building toxicity concentration curve. Two optimal concentrations, 1 and 2 uM, of cytarabine were selected for experiments with Wip1 chemical inhibitor GSK2830371 (Selleckchem, Houston, TX, USA). Cells viability after drug exposure was measured with the use of XTT proliferation kit. Cell cycle was measured by flow cytometry with propidium iodide. Apoptosis was analyzed by FACS on CytoFLEX flow cytometer with AnnexinV kit. Western blots were performed according standard protocol. Antibodies against Wip1, were purchased from Santa Cruz (Clone F10, Paso Robles, CA, USA), LC-3, p53 were purchased from Cell Signaling (Ab-6, London, UK).

**Results:** The treatment with cytarabine of two AML cell lines, OCI-AML2 (AML FAB M4) and Mono- Mac1 (AML FAB M5) induced delayed cell cycle progression and proliferation of tumor cells but was not able to induce a massive cell death due to the ability of cytarabine to induce autophagy. As reported previously, cytarabine reduced the phosphorylation of the major negative regulator of autophagy, the mammalian target of rapamycin, mTOR. Inhibition of Wip1 phosphatase with a specific chemical inhibitor, GSK2830371, led to the shift from autophagy towards programmed cell death, apoptosis in OCI-AML2, but not in Mono-Mac1 cell line. We verified the levels of Wip1 in both cell lines and found that OCI-AML2 has high levels of Wip1 protein. On contrary, we were not able to detect any Wip1 expression in Mono-Mac1 AML cells that may explain the absence of GSK2830371 effect on cell death in those cells.

**Conclusions:** We conclude that Wip1 phosphatase could be a new target in the treatment of AML and its inhibition potentiate the cytarabine efficiency in inducing cell death of AML cells, possibly by blocking autophagy and by favoring apoptosis. At the same time, Wip1 levels in AML cells could be a prognostic marker determining the efficiency of the proposed anti-tumor strategy.

**Funding:** The research was supported by grant from the Russian Science Foundation 19-75- 20128.

**Disclosure:** The authors confirm that they have no conflict of interest.

## 9

### Ionophore nigericin as potential agent for targeted elimination of senescent endometrial stromal cells

#### Anastasiia Griukova^1^, Pavel Deryabin^1^, Alla Shatrova^1^, Aleksandra Borodkina^1^

##### ^1^Institute of Cytology of the Russian Academy of Sciences, Saint-Petersburg, Russia; ORCID: 0000-0001-5283-4144

**Introduction:** Aging is one of the main risk factors for progression of various diseases, including different types of cancer, cardiovascular and neurodegenerative disorders. The accumulation of senescent cells in tissues is considered to mediate the impairment of their normal functioning contributing to aging of an organism. In this regard, the search and development of senolytics, compounds that selectively kill senescent cells, is one of the strategies to fight age-related diseases. For now, there are some examples of the successful application of such agents both in vitro and in vivo. The Results. of our research group indicate that senescence of endometrial stromal cells (eSCs) may mediate the endometrium dysfunction. We hypothesize that targeted removal of senescent cells may serve as an approach to prevent their negative impact on the properties of endometrial tissue. Thus, our investigation aimed to find an agent for the selective elimination of senescent eSCs.

**Materials and methods:** Viability and different parameters of young and senescent eSCs were assessed by FACS while the signaling pathways were detected by Western blotting.

**Results:** Senescent cells are known to possess increased stress resistance. In this regard, initially, we checked whether the senescent eSCs have an enhanced survival compared to the young ones. Indeed, senescent eSCs appeared to be more resistant to all the treatments tested: oxidative stress, heat shock, DNA damage and apoptosis induction. At the next stage, we evaluated various parameters of young and senescent cells in order to find the distinctive features of the last ones. As a result, drop in intracellular pH, depolarization of the plasma membrane, decrease in the mitochondrial membrane potential as well as impairment of autophagic flux were revealed in senescent eSCs. Therefore, further we aimed to select compounds, which could affect these “impaired” characteristics of senescent cells to make them incompatible with survival. We tried to influence each of the listed parameters using different agents, but none of them resulted in the selective death of senescent eSCs. Thereby, we decided to test a complex impact on the disturbed features of senescent cells and applied a well- known ionophore nigericin chosen as a compound capable of such an influence. As expected, nigericin led to the decrease in intracellular pH, depolarization of the plasma membrane, hyperpolarization of the mitochondrial membrane potential as well as blocked the autophagic flux. Moreover, nigericin treatment was more specific to eliminate senescent eSCs than younger ones.

**Conclusion:** We demonstrated the enhanced stress resistance of senescent eSCs compared to the young cells. In search of distinctive features of senescent eSCs we revealed their several “impaired characteristics”. However, only complex impact of ionophore nigericin selectively killed senescent eSCs. Therefore, we can conclude that nigericin may be considered as potential agent for targeted elimination of senescent cells from endometrial tissue.

**Funding:** This study was funded by the Russian Science Foundation (# 19-74-10038).

**Disclosure:** The authors confirm that they have no conflict of interest. The work was funded by the Russian Science Foundation (# 19-74-10038).

## 10

### Autophagy genes expression profiling in secondary glioblastoma cells transcriptome

#### Natalya Gubanova^1^, Anatoly Bragin^2^, Gennady Vasiliev^1^, Vladimir Babenko^1^, Mikhail Ponomarenko^1^, Yuriy Orlov^1,3^

##### ^1^Institute of Cytology and Genetics, Siberian Branch of the Russian Academy of Sciences, Novosibirsk, Russia; ^2^Novosibirsk State University, Novosibirsk, Russia; ^3^Institute of Digital Medicine, I.M. Sechenov First Moscow State Medical University, Moscow, Russia; ORCID: 0000-0003-2940-3287

**Introduction:** Glioblastomas are the most common type of brain tumor and often grow rapidly with a poor prognosis for the patient. Tumor cells hijack cellular processes, which leads to resistance to radio- and chemotherapy. Recent investigations have demonstrated a controversial role for autophagy in the induction of this resistance. In the current study, we have analyzed the expression of auto- and mitophagy genes in primary cell cultures of normal brain and secondary glioblastoma obtained on a patient following treatment with radio- and chemotherapy.

**Materials and methods:** The samples of secondary glioblastoma and normal brain were obtained on patients of the same age and sex. Before surgical resection, the patient with secondary glioblastoma was treated with temozolomide and radiotherapy. Cell culture has been isolated and propagated under the same conditions, which made it possible to avoid changes in gene expression caused by the individual characteristics of patients as well as pathological changes in blood vessels and brain tissues. RNA-seq sequencing of cell samples was done using Illumina HiSeq 1500. Cufflinks program was used to assess gene expression and find differently expressed genes.

**Results:** Comparison of expression profiling of glioblastoma cell culture and normal brain showed that 78 out of 137 genes involved in the regulation of autophagy were expressed differentially. Most of the genes (57 out of 78) have reduced expression compared to the normal brain. The expression of the PIK3CD, CFLAR, RRAS, MAPK3, ULK1, DDIT4, ATG9B, BNIP3, MTMR4, PRKAA2, RRAGD, VAMP8 genes is decreased 2 fold and more. At the same time, the overexpression 2 fold and more is observed in the PIK3R1, IRS1, PIK3R3, BCL2, RAB7B genes.

Analysis of expression profiling of genes involved in mitophagy showed similar Results. Of the 68 genes, 40 were differentially expressed, while for 25 of them the expression was suppressed in tumor cells. The expression levels of BNIP3L, RRAS, ULK1, ATG9B, PINK1, BNIP3, MITF, OPTN, genes were decreased 2 fold and more. Genes AB7B and E2F1 were overexpressed in tumor at the same ratio.

**Conclusions:** The observed decrease in the expression of genes necessary for auto- and mitophagy reduces the intensity of these processes and causes the resistance of glioblastoma cells to chemotherapy and radiation therapy.

**Disclosure:** The authors confirm that they have no conflict of interest. Data available upon reasonable request to the corresponding author.

## 11

### Targeting autophagy as means to eliminate tumor cells resistant to HER2 inhibitors

#### Anastasia Gudovich^1^, Alexandra Daks^1^, Oleg Shuvalov^1^, Evgenii Smirnov^1^, Olga Fedorova^1^

##### ^1^Institute of Cytology, Russian Academy of Sciences, Saint-Petersburg, Russia; ORCID: 0000-0002-8210-1045

**Introduction:** Breast cancer is the most common cancer in women worldwide. Every fifth case of breast cancer is characterized by high expression of the HER2 protein. Being a tyrosine kinase receptor, HER2 is defined as a prognostic and predictive marker of disease. HER2-positive breast cancer is characterized by a more aggressive form of the disease. Also, patients with HER2 overexpressing tumors tend to have the worst overall survival rates.

Pirh2 (also known as RCHY1) is the RING-type E3 ubiquitin ligase that promotes the ubiquitin-mediated proteasomal degradation of different proteins including p53. Currently, Pirh2 is deemed to play both oncogenic and tumor-suppressive roles in cancer. The aim of our study was to determine the effect of E3 ligase, Pirh2, on the sensitivity of HER2-positive breast cancer cells to HER2 inhibitors.

**Materials and methods:** HER2-positive breast cancer cell lines BT-474 and SK-BR-3 with different expression status of Pirh2 (control and knockdown) were used. The MTT assay was used to measure cell viability. Chloroquine diphosphate (CQ) was used as inhibitor of autophagolysosomes formation. The level of autophagy level was analyzed by the level of LC3 and p62 proteins and Lysotracker Red staining.

**Results:** Kaplan-Meier plotter data analysis showed the relationship between level of Pirh2 expression and prognosis of patients with HER2-positive breast cancer. Low expression of Pirh2 was associated with poor survival in HER2-positive breast cancer patients. Using MTT-assay we have shown that suppression of Pirh2 expression leads to cell resistance to afatinib and neratinib.

There are several evidences that autophagy mediates drug resistance. Moreover, autophagy has become a promising target for anti-cancer therapy. We studied the influence of CQ to overcome resistance to Her2 inhibitors in cancer cell lines with knockdown of Pirh2. Cell viability assays showed that the combined treatment of cells with CQ and inhibitors of Her2 led to diminished cell survival both in control and knockdown of Pirh2 cell lines. We also examined the level of autophagy in these cell lines by western blotting for LC3-II and staining with Lysotracker Red.

**Conclusions:** We hypothesize that Pirh2 is involved in the regulation of autophagy and using inhibitors of autophagy can help to overcome primary resistance in cell lines with low level of expression Pirh2.

**Funding:** This work was supported by RSF research project 18-75-10076.

**Disclosure:** The authors confirm that they have no conflict of interest. This work was supported by RSF research project 18-75-10076.

## 12

### Set7/9 sensitizes NSCLC cells to genotoxic therapy through downregulation of autophagy and activation of apoptosis

#### Matvey Ivanov^1^, Alexandra Daks^1^

##### ^1^Institute of Cytology of the Russian Academy of Sciences, Saint-Petersburg, Russia; ORCID: 0000-0003-0495-1244

**Introduction:** Set7/9 is a lysine methyltransferase (KMTase) that was first described as the enzyme that methylates the lysine 4 of histone H3 and a number of lysine residues of histone H1.4. Later it was shown that Set7/9 is responsible for the transfer of a methyl group to lysine residues of various non-histone substrates involved in the regulation of various cellular processes, such as cell cycle control, differentiation, response to DNA damage, and chromatin alteration. Set7/9 has also been shown to methylate the ATG16L1 protein involved in autophagosome formation, thereby inhibiting autophagy.

**Materials and methods:** Non-small-cell lung cancer (NSCLC) constitutes 85% of all lung cancers, and is the leading cause of cancer-related death worldwide. Despite the significant progress in anticancer therapy development, the research on lung cancer mechanisms and approaches of effective treatment is still undoubtedly relevant. In a large number of cases, NSCLC is characterized by mutations in the TP53 gene. One of the key functions of p53 transcription factor is to control the expression of the pro-apoptotic gene such as Bax, Bim, Noxa, and PUMA. The depletion or mutation of p53 causes more aggressive course of the disease and poorer prognosis for NSCLC.

**Results:** We used H1299 cell line to study the role of Set7/9 in autophagy regulation and cell response to the genotoxic drugs treatment. H1299 is the human non-small cell lung carcinoma cell line with partial deletion of TP53 gene, and lack expression of p53 protein. As a result, we demonstrated that both knock-down of Set7/9, and inhibition of its methyltransferase activity via specific inhibitor R-PFI-2, leads to downregulation of autophagy. We also demonstrated that H1299 with Set7/9 knock-down (Set7/9KD) demonstrated the increased sensitivity to doxorubicin and etoposide treatment. This effect was caused by increased apoptosis level in Set7/9KD cells compared to the wild-type control H1299 cells. We also observed the decrease in autophagy level in the genotoxic stress conditions that is consistent with the paradigm of mutual suppression of apoptosis and autophagy.

**Conclusions:** Thus, we conclude that using Set7/9 inhibitors as a part of combined anticancer therapy can be considered as a potentially promising strategy for NSCLC treatment.

**Funding:** This work was supported by Russian Science Foundation Grant No. 19-75-10059.

**Disclosure:** The authors confirm that they have no conflict of interest. This work was supported by Russian Science Foundation Grant# 19-75-10059.

## 13

### Bafilomycin A1 induces different cell death types in non-senescent and senescent Ras-transformed cells

#### Elena Kochetkova^1,2^, Olga Bystrova^1^, Marina Martynova^1^, Svetlana Zubova^1^, Tatiana Bykova^1^, Valery Pospelov^1^, Tatyana Pospelova^1^

##### ^1^Institute of Cytology, of the Russian Academy of Sciences, St.Petersburg, Russia; ^2^Karolinska Institute, Dept. of Physiology and Pharmacology, Sweden; ORCID: 0000-0002-6897-7171

**Introduction:** The modern strategies of tumor cell elimination are linked with searching for targets, which would result in cell death. Many death-inducing agents promote cell death through targeting autophagy. We have shown, that several agents that target mitochondria directly or indirectly, cause two types of death in Ras-transformed malignant cells—apoptotic and autophagic death. Bcl2 mimetic ABT199, MEK/ERK kinase inhibitor PD0325901, mTORC1 inhibitor pp242 induce mitochondria damage. Upon treatment with ABT199, PD0325901, low doses of pp242 cells demonstrate mitochondria damage, but autophagy activated is able to remove damaged organelles and restore cellular viability. Treatment with high doses of mTORC1 inhibitor pp242 Results. in massive mitochondria damage that cannot be reduced by autophagy, and eventually, in apoptotic cell death. Senescent cells have specific metabolic phenotype and are presumed to be resistant to apoptosis upon serum and amino acid deprivation. We aimed to define, how senescent Ras-transformed cells would respond to mTORC1 suppression by pp242, or to lysosome suppression by lysosomal vATPase inhibitor Bafilomycin A1 (Baf).

**Materials and methods:** Senescent cells are characterized with hypertrophic phenotype and secretion of various factors, which form Senescence-Associated Secretory Phenotype (SASP). Autophagic flux in senescent cells is decreased in comparison with non-senescent ones. We have found that senescent Ras-transformed cells exposed to low doses of mTORC1 inhibitor pp242 activate autophagy as a result of mTORC1 suppression and mitochondria damage. However, pp242-induced autophagy in senescent cells ceases after 24h of treatment, but cells isolate damaged mitochondria in huge LC3- negative vacuoles, that are excreted later. The origin of vacuoles observed is indefinite, the transmission electron microscopy and immunofluorescence show accumulation of vacuoles followed by direct secretion of cargo. Possibly, these vacuoles are linked with secretory system of senescent cells. This mechanism becomes involved in the removal of damaged organelles and restoration of cellular viability. Attenuation of autophagy promotes accumulation of damaged organelles and aberrant proteins, what, for example, is characteristic of neurodegenerative diseases. Understanding the possible alternative mechanisms may provide new strategies for the therapy of such diseases.

**Results:** Both rodent and human Ras-transformed cell lines exposed to Baf demonstrate massive cell death, with almost 10-fold decrease of viability (according to MTT test and clonogenic assay). Baf treatment decreases lysosomal acidification, as follows from data with Lysotracker Green staining. Surprisingly, Bafilomycin induces drastic mitochondria damage. Transmission electron microscopy of Baf-treated non-senescent cells shows accumulation of huge empty cavities. Eventually, cells undergo apoptosis. In senescent human cells, exposed to Bafilomycin, cytoplasm is filled with numerous vacuoles, but these vacuoles contain damaged organelles. However, the cargo is not degraded, probably, due to suppression of lysosomal pH, and cells cannot restore their viability. Though senescent Baf-treated cells isolate damaged organelles in huge vacuoles, the degradative and secretion systems in Baf-treated cells are attenuated, and the vacuoles accumulate, filling all cytoplasm of hypertrophic cells.

**Conclusions:** While non-senescent Baf-exposed cells undergo apoptosis, senescent cells undergo non-apoptotic undefined death mechanism, as follows from DNA fragmentation and the clonogenic data. Our data show that Baf allows to eliminate senescent Ras-transformed cells with high efficacy through induction of alternative, non-apoptotic death program.

**Disclosure:** The authors confirm that they have no conflict of interest.

## 14

### Methods for treating glial tissue tumors

#### Galina Kosyakova^1,2^, Ilia Kazurov^2^, Vasilina Mikhailova^2^, Natalia Mikhailova^3^, Veronika Novak^3^, Oleg Ostreiko^3^

##### ^1^The Institute of Experimental Medicine, St.Petersburg, Russia; ^2^Saint-Petersburg State Chemical Pharmaceutical University, St.Petersburg, Russia; ^3^Department of Biotechnology SIC at the Research Institute of Hematology, Oncology and Transplantology them. R.M. Gorbachevoy in St. Petersburg, St.Petersburg, Russia; ORCID: 0000-0001-7211-7839

**Introduction:** Glioblastoma multiform is the most lethal form of brain tumors. Upon initial diagnosis of glioblastoma multiform (GBM), standard treatment consists of maximal surgical resection, radiotherapy, and concomitant and adjuvant chemotherapy with temozolomide. Median time to recurrence after proper use of these methods is 6.9 months. New approaches to design optimized therapies are widely used in patients with GBM. Two of the most investigated variants of minimally invasive methods are the laser interstitial thermotherapy (LITT) and the high-intensity focused ultrasound (HIFU) therapy. These techniques are based on the principle of cell death originating due to the temperature rise. To study efficiency and safety of the LITT and HIFU therapy pilot study utilizing the subcutaneous rat C6 glioma model was performed.

**Materials and methods:** Glioma C6 culture cells in concentration 6 × 10*6/μL were injected into the left flank of 2-month-old Wistar male rats, 250–300 g. During a 12 days’ time period the glioma of 12–20 mm in diameter developed in the injection site in all animals. The rats were divided into three groups: the control group (no treatment group for accessing the dynamics of tumor growth), the HIFU therapy group (Pulsed mode, US frequency = 2,2 MHz), the LITT group (continuous laser with 970 nm wavelength). Then, 2 days later, the measurement of diameter of the subcutaneous tumor of each rat was performed, and bone marrow was taken, and blood smears were made.

**Result and discussion:** The measurement of subcutaneous tumors after treatment with HIFU or LITT showed the reduction of tumor tissue volume. Whereas in control group no significant change of tumor site diameter could be seen. In blood samples of control group rats increased level of micronuclear erythrocytes level was insignificant. Therefore, minimally invasive methods of glioma treatment are effective and safe alternatives of surgical resection. The advantages of these treatment methods include the possibility of subtotal resection of deep inaccessible gliomas, the minimal tissue injury in the surgical approach site, which allows for less duration of postoperative patient’s hospital stay.

**Disclosure:** The authors confirm that they have no conflict of interest.

## 15

### Analysis of autophagy in the hippocampus of audiogenic rats during epileptogenesis

#### Aleksey Kulikov^1^, Margarita Glazova^1^, Elena Chernigovskaya^1^

##### ^1^Sechenov Institute of Evolutionary Physiology and Biochemistry of the Russian Academy of Sciences, St.Petersburg, Russia; ORCID: 0000-0003-3453-5030

**Introduction:** Autophagy is a normal process of elimination of cellular structures in response to different forms of stress, it plays a critical role in cellular homeostasis and is highly involved in the formation of neural circuits, synaptogenesis, and neurogenesis. It was shown that autophagy impairments are associated with epilepsy. The hippocampal formation is one of the most affected areas, where epileptogenesis is usually associated with dramatic morphological and functional changes. There are different laboratory rodent models of hereditary epilepsy, and one of which is Krushinsky–Molodkina (KM) rats genetically prone to audiogenic epilepsy. Initially, audiogenic seizure (AGS) expression is based on activation of the midbrain structures, but multiple AGSs induce overspread of epileptiform discharges through limbic structures that can be considered as a model of limbic epilepsy.

**Objective:** In our study, we investigated the effects of repetitive AGSs on autophagy in the hippocampus of KM rats.

**Materials and methods:** Male and female adult KM rats were used in the experiments. Seizure-induced alterations of autophagy were analyzed in the hippocampus of KM rats after 4, 7, and 25 AGS expression. Naïve KM rats were used as a control. AGS was induced by sound stimulation at 10 kHz and 50 dB once per day. The hippocampi were collected either 24 h or in a week after the last AGS. Half of the animals (*n* = 5 for each group) were used for immunofluorescent analysis. Histological sections containing the hippocampus were stained for LC3B and cathepsin D. Another half of animal (*n* = 5) were used for Western blot analysis. The protein extracts were immunoblotted with affinity-purified antibodies against LC3B, Beclin-1, and p62. Statistical analysis was performed with a nonparametric Kruskal–Wallis test using GraphPad Prism 7.

**Results:** It has been shown that 4 AGS led to an increase in LC3BI and LC3BII expression in the hippocampus of KM rats, but p62 expression was not changed in comparison with control. Seven days after the fourth AGS the expression of studied autophagy proteins was not altered.

7 AGS did not affect the expression of Beclin-1, LC3BI, and LC3BII in the hippocampus, but p62 expression was significantly decreased. However, in a week after 7 AGS we observed an increase in Beclin-1 and LC3BII and a decrease in p62 and LC3BI. Moreover, the immunofluorescent analysis revealed a significant increase in the number of cells which co-expressed LC3B (autophagosome marker) and cathepsin D (lysosome hydrolyze) in the CA4 field of the hippocampus. Additionally, after

7 AGS the entire cell population in the granular layer of the Dentate Gyrus examined by DAPI fluorescence was significantly lower. But one week recovery period led to the restoration of the number of cells to control.

25 AGS did not alter the expression of autophagy proteins (Beclin-1, p62, LC3BI, and LC3BII), but the entire cell population in the granular layer, the hilus, and CA4 was significantly lower and did not restore after a recovery period.

**Conclusions:** We demonstrated that 4 AGS expression did not induce autophagy in the hippocampus. However, 7 AGS led to the initiation of autophagy that probably after a week recovery period promoted the restoration of cell loss induced by repetitive AGS. On the other hand, audiogenic kindling induced by 25 AGS was finalized by significant cell loss in the granular layer but did not affect autophagy. Our data revealed that activation of autophagy during the early stages of epileptogenesis has a neuroprotective effect. Thus, we can hypothesize that activation of autophagy by special treatments at status epilepticus might induce neuroprotective mechanisms.

**Funding:** The study was supported by RFBR 19-015-00070.

**Disclosure:** The authors confirm that they have no conflict of interest. The study was supported by RFBR 19-015-00070.

## 16

### Nuclear lamina in dermal fibroblasts in patients with breast cancer and people at risk of developing cancer

#### Mirya Kuranova^1^, Alexandra Nozdracheva^2^, Nadezhda Pleskach^1^

##### ^1^Institute of Cytology of the Russian Academy of Sciences, St.Petersburg, Russia; ^2^Peter the Great St.Petersburg Polytechnic University (SPbPU), Russia; ORCID: 0000-0002-2778-3629

**Introduction:** The nuclear lamina is an important element of the cell nucleus and is involved in the organization of chromatin, DNA replication and supports the nuclear membrane. It plays an important role in mitosis, apoptosis and autophagy, during nuclear breakdown.

**Objective:** Using the method of indirect immunofluorescence, we investigated the state of the nuclear lamina in the cell lines of dermal fibroblasts of patients with breast cancer 30 and 55 years old (BC30SP and BC55SP). The second group of cell lines studied was a group of patients at risk of developing cancer: a patient line with a 5382incC mutation in the BRCA1 gene, 30 years old (BRCA1SP) and two lines of mothers (45 and 47 years old) of patients with a severe hereditary disease ataxia-telangiectasia (AT), which is associated with a high risk of developing cancer (AT8MSP and AT9MSP). Dermal fibroblast cell lines from healthy women of 30 and 55 years old (N9SP and FK19) were selected as healthy donors. All cell lines were obtained in the laboratory of radiation cytology of the Institute of Cytology of the Russian Academy of Sciences. Cell lines were stained with LMNA A / C antibodies (Abcam, UK). Microscopy was performed using a confocal microscope Leica Microsystems.

**Materials and methods:** We looked for such features of the nuclear lamina as the presence of both blebs and invaginations, which are considered a sign of cell aging, as well as fragmented parts of the lamina, that is a marker of the onset of autophagy process. The degree of depletion of the nuclear lamina (the presence of a characteristic rim), was also assessed. In each cell line, 300 cells were analyzed.

**Results:** Our Results. show that the smallest percentage of the simultaneous presence of blebs and invaginations of the nuclear lamina was present in the cells of a healthy donor of 30 years (3.2%), then in the cells of a healthy donor of 55 years (4.6%). Slightly higher percentage of blebs and invaginations was demonstrated by cells from the line of a patient with breast cancer 30 years old (BC30SP)—5.41% and from the line of the mother of a patient with AT (AT9SP)—5.9%. In the cell line of the mother of the second patient with AT (AT8SP), the percentage of blebs and invaginations of the nuclear lamina was 9.3%. The highest percentage of the simultaneous presence of blebs and invaginations of the nuclear lamina was demonstrated by the cells of the patient lines with the 5382incC mutation in the BRCA1 gene (BRCA1SP)—24.55% and the line of the patient with breast cancer 55 years old (BC55SP)—16.3%.

**Conclusions:** Interestingly, the percentage of blebs and invaginations of the nuclear lamina in the cells of a 55-year-old breast cancer patient was 3 times higher than in the cells of a younger patient with breast cancer. Cells with nuclear fragmentation, in which the process of autophagy had begun, were also found in the cell lines of healthy donors of 30 and 55 years old and made up 1% and 2% of the cell population. The highest percentage of cells with signs of autophagy was shown in the lines of a 30- year-old patient with breast cancer and a patient with 5382incC mutation and was 6.7% and 5%, respectively. In the line of a 55-year-old patient with breast cancer, the percentage of cells with a fragmentary nucleus was 2.3%. In the lines of mothers of patients with AT8SP and AT9SP, the percentage of cells with signs of autophagy was 1.3% and 1.5%, respectively.

Interestingly, the percentage of cells with signs of autophagy was also 3 times higher in the cells of a younger patient with breast cancer than in a 55-year-old patient. The presence of a characteristic rim of the nuclear lamina, which is an indicator of its thinning, was demonstrated only by the cells of mothers of patients with AT.

**Funding:** This work was supported by the Council for Grants of the President of the Russian Federation MK-3638.2019.7.

**Disclosure:** The authors confirm that they have no conflict of interest. This work was supported by the Council for Grants of the President of the Russian Federation MK-3638.2019.7.

## 17

### Increased level of NF-kappaB affects chemosensitivity and transcription of SASP related genes in lung cancer cells H1299

#### Ekaterina Lomert^1^, Elizaveta Zhaivoron^1^, Ksenia Novitskaya^1^, Daria Kriger^1^, Dmitri Tentler^1^

##### ^1^Institute of Cytology of the Russian Academy of Sciences, St. Petersburg, Russia; ORCID: 0000-0003-3732-0553

**Introduction:** The evolutionarily conserved transcription factor NF-kappaB plays an important role in inflammation, immune response, and in almost all aspects of cellular activity. It is constitutively activated in many types of cancers, which leads to the expression of various target genes, and subsequently to malignant transformation, abnormal cell proliferation, metastases, and chemoresistance. In addition, NF-kappaB is also involved in autophagy control either directly via regulation of the pro-autophagic Beclin1 gene expression or indirectly by modulating other autophagy inducing pathways.

**Materials and methods:** A number of studies showed prognostic significance of NF-kappaB expression level for the outcome of patients with non-small cell lung cancer (NSCLC). To model constitutive NF-kappaB activation in NSCLC cells we generated a stable H1299 cell line with elevated level of the major activating NF-kappaB subunit RelA. The *RELA* gene expression in the H1299/RelA cells was increased 2.5 times comparing to control cell line. Increased expression of NF-kappaB target genes *IKBA* and *ICAM1* demonstrated the functional activity of the exogenous RelA. Elevated RelA level inhibited cell proliferation but increased the migration rate. To reveal the effects of *RELA* expression on sensitivity to genotoxic drugs, we treated the cells with different concentrations of doxorubicin and etoposide for three days. The calculated IC50 showed that H1299/RelA cells were more resistant to etoposide than control cells while sensitivity to doxorubicin was not affected. Both cell lines undergo G2/M cell cycle arrest. To determine possible reason for the decreased proliferation rate but increased etoposide resistance of H1299/RelA cells, we checked the expression of senescence-associated secretory phenotype (SASP) genes. The genes *CDKN1A, IL1A*, *IL1B, IL6, TNF, PTGS2, INOS, CCL2* were found to be upregulated in H1299/RelA cells.

**Results and conclusions:** We suggest that elevated RELA expression may increase H1299 resistance to etoposide by stimulating senescence, which also leads to more aggressive phenotype along with the increased migration rate. In parallel, high RELA levels may induce cytoprotective autophagy and anti-apoptotic pathways as an additional mechanism of decreased cell sensitivity to etoposide treatment. This hypothesis is to be tested next.

**Funding:** This work was supported by the grant from the Russian Government 14.W03.31.0029.

**Disclosure:** The authors confirm that the project was supported by the Grant from Russian Government (Mega Grant) 14.W03.31.0029.

## 18

### Generating tp53 mutant MCF7 cell line with CRISPR/cas9: cell model for targeted therapies

#### Regina Mirgayazova^1^, Rania Khadiullina^1^, Rimma Mingaleeva^1^, Matthias Baud^2^, Albert Rizvanov^1^, Emil Bulatov^1^

##### ^1^Kazan Federal University, Kazan, Russia; ^2^University of Southampton, Southampton, UK; ORCID: 0000-0003-2961-0032

**Introduction:** The tumor suppressor p53 plays a key role in a variety of signaling pathways that control the cell cycle and are responsible for the stability of the human genome. The *TP53* gene is the most commonly mutated gene in human cancer with mutations occurring in about half of all cancer cases. The presence of *TP53* mutation can significantly worsen the prognosis of cancer patients mainly due to metabolic changes in tumor cells. Evaluating the effect of targeted cancer therapies on tumors with various mutations can become a challenge due to a vast variety of point mutations and limited availability of commercial cell lines with such mutations. Therefore, employing genome-editing tools, *e.g*. CRISPR/Cas9, to generate cell lines with the required *TP53* mutations presents a promising new way to assess the drug efficacy.

The Y220C missense mutation is the ninth most common for *TP53* gene and is annually observed in approximately 100,000 new cases of diagnosed cancer worldwide. In this study we are developing genome-editing approaches based on CRISPR/Cas9 to generate MCF7 cell line with Y220C mutation in *TP53* gene.

**Materials and methods:** In this project we used a range of CRISPR/Cas9-based tools to obtain a *TP53* mutant cell line: lentiviral transduction, CRISPR Base Editing and Prime Editing. Golden Gate cloning was used to create plasmid encoding pegRNA for prime editing. The presence of Y220C mutation in generated cell lines was confirmed by sequencing. The expression of p53 protein (mutant and wild-type) was confirmed by immunoblotting. The previously developed small molecule compounds specifically targeting mutant p53(Y220C) protein were used as prospective anticancer therapeutics in MTS cell viability assay.

**Results and conclusions:** We have successfully generated MCF7 (*TP53-Y220C*) cell line with lentiviral transduction, the presence of the mutation was confirmed by sequencing. The experiments are ongoing to employ CRISPR Base Editing and Prime Editing to obtain MCF7 (*TP53-Y220C*) from MCF7 (*TP53* wild-type). Sequencing is expected to confirm the presence of monoclones with *TP53- Y220C* mutation.

**Funding:** The study was supported by the Russian Science Foundation grant 19-74-10022 to E.B.; R.M. thanks RFBR 19-54-10005.

**Disclosure:** The authors confirm that they have no conflict of interest. The study was supported by the Russian Science Foundation grant 19-74-10022. Data avaliable upon reasonable request to the corresponding author.

## 19

### Role of cystamine in autophagy of breast cancer cells with different status of p53

#### Sergey Parfenyev^1^, Alexandra Daks^1^, Olga Fedorova^1^, Nickolai Barlev^1^

##### ^1^Institute of Cytology of the Russian Academy of Sciences, St-Peterburg, Russia; ORCID: 0000-0002-0531-4389

**Introduction:** Autophagy is a regulated pathway involving a lysosomal degradation of cytoplasmic organelles and components. However, autophagy is currently considered not only as a degradation process but also as a cellular mechanism necessary to maintain cell homeostasis. Disruption of autophagy is directly linked to a number of diseases including cancer. Cystamine is a radioprotective agent used in the treatment of malignant tumors. Cystamine is also used to inactivate transglutaminase 2 (TG2) enzyme, which promotes the triggering of autophagy in cells with expression of the wild-type tumor suppressor p53 protein.

The aim of this work was to study the effect of the TG2 inhibitor on the level of autophagy in breast cancer cells with different p53 status.

**Methods:** Breast cancer cell lines MCF7 and MCF7-shp53 were used in the work. p53 was knocked down in MCF7-shp53 cells by stable infection with the lentiviral construct containing p53-specific hairpin. Cells were treated with cystamine (200 µM/L) for 14 h. Chloroquine (CQ) was added for 14 h at a concentration (50 µM/L). Autophagy markers LC3-II and p62 were detected by Western blot analysis.

**Results:** We found that after treatment with the autophagy inhibitor CQ the level of proteins LC3-II and p62 increased in MCF7 cells. In contrast, in MCF7-shp53 the level of the main autophagy markers LC3-II and p62 changed subtly suggesting an important role of p53 in activating the autophagy. The addition of cystamine led to a significant decrease in the level of autophagy in both cell lines regardless of the p53 status which indicates the presence of a p53-independent pathway for the regulation of TG2-dependent autophagy in breast cancer cells.

**Conclusions:** P53 is involved in the regulation of the onset of autophagy in breast cancer cells. Cystamine-induced suppression of TG2 activity leads to a decrease in the level of autophagy in MCF7 breast cancer cells regardless of p53 status.

**Funding:** This work was supported by the Russian Science Foundation Grant No. 19-45-02011.

**Disclosure:** The authors confirm that the project was partly supported by the the Russian Science Foundation Grant No. 19-45-02011.

## 20

### The role of autophagy in overcoming cellular senescence of human MSC in 3D-2D model

#### Irina Neganova^1^, Arina Saveleva^1^, Polina Fedyukina^1^, Olga Bystrova^1^, Marina Martynova^1^, Ekaterina Baidyuk^1^

##### ^1^Institute of Cytology of the Russian Academy of Science, Saint Petersburg, Russia; ORCID: 0000-0001-5712-2170

**Introduction:** Human MSCs (hMSCs) play an important role in the repair of various injuries of organs and tissues of the human body. They are characterized by active proliferation, the ability to self-renewal, differentiation in osteo-, chondro- and adipogenic directions, regulation of angiogenesis, as well as strong immunomodulatory properties and the secretion of the anti-inflammatory molecules. All these qualities make it possible to use hMSCs in regenerative medicine. However, the active use of these cells is limited, because MSCs undergo cellular senescence in vitro—a decrease in the functional activity and cell proliferation as the number of cell passages increases. It was shown that late passage hMSCs after passing through the “3D spheroids” phase restore the ability to actively proliferate and have a typical phenotype of early cells. However, studies of the 2D-3D-2D model of hMSCs mainly were dedicated to investigation of the enhanced anti-inflammatory effect of these cells, changes in their secretome, and higher survival. Our aim is to determine the cellular mechanisms and signaling pathways important and responsible for the obtained effect in the 2D-3D-2D model.

**Materials and methods:** We used hMSCs of two lines (obtained from the adipose tissue of healthy adult individuals), which were provided by the “Pokrovsky Bank of Stem Cells”, as well as fetal hMSCs obtained earlier at the INC RAS. 3D spheroids were prepared from late passage cells positive for the main markers of cellular senescence, using the hanging drop technique. The cell cycle and activation of autophagy in 3D spheroids were compared with the proliferative activity of 2D cultured cells of early and late passages, as well as with these characteristics in cells after leaving the 3D spheroid. Obtained data correlate with Results. from other groups and confirm the overcoming of cellular aging of hMSCs in the 2D-3D-2D model. In addition, after leaving 3D and going back to 2D culture condition, hMSCs retained a normal karyotype and a high level of surface markers expression.

**Results and conclusions:** Electron microscopic methods together with immunofluorescent (IF) staining for p62 (receptor of autophagy, deliver ubiquitinated proteins to the autophagosome for degradation), p230 (marker of trans-Golgi network) and LC3 (marker of autophagosome), confirmed the activation of the autophagy process in 3D spheroids of hMSC in comparison to 2D cultures of early passages. It is known that one of the key regulators of autophagy is the serine-threonine kinase mTOR. Currently, we are investigating the role of mTOR in our model. Due to the active role of lysosomes in the autophagy process, we used LysoTrackerRedDND-99 to find out if lysosomes accumulate on late passages and spheroids. The Results. showed that the number of lysosomes increased 135 times on late passages and 70 times in 3D spheroids compared to the early passages. Thus, our data suggest activation of the autophagy process in 3D spheroids as the main mechanism for overcoming cellular senescence of hMSC sin 2D-3D-2D model.

**Funding:** The work was carried out within the framework of the RFBR grant No. 20-015-00060 and with the support of the RF Government grant No. 14.W03.31.0029.

**Disclosure:** The authors confirm that the project was partly supported by the Grant from Russian Government (Mega Grant) 14.W03.31.0029.

## 21

### Autophagy in the human retina in glaucoma

#### Natalia Obanina^1,2^, Nataliya Bgatova^1^

##### ^1^Research Institute of Clinical and Experimental Lymphology – Branch of the Institute of Cytology and Genetics, Siberian Branch of the Russian Academy of Sciences, Novosibirsk, Russia; ^2^Novosibirsk State University, Novosibirsk, Russia; ORCID: 0000-0002-4507-093X

**Introduction:** To identify features of the ultrastructure of the human retina in the terminal stage of glaucoma.

**Materials and methods:** The object of the study was retinal fragments of medically enucleated eyes of patients with a diagnosis of “terminal stage of glaucoma” of the Novosibirsk branch of Аcademician S.N. Fyodorov Federal State Institution «Intersectoral Research and Technology Complex “Eye Microsurgery”. All studies were carried out with the permission of the Bioethical Committee of the Novosibirsk branch of the IRTC “Eye Microsurgery”. The written informed consent of the patients to study the biological material was obtained. Retinal specimens were prepared for transmission electron microscopy according to the standard method and studied in the electron microscope JEM 1400. Morphometric studies were performed in accordance with generally accepted principles using the computer program Image J (Wayne Rasband, Kensington, MD, USA).

**Results:** The ultrastructure of cells of various layers of the retina was explored: the layer of rods and cones, the outer nuclear and the outer plexiform layers. Structural signs of organelle damage and the development of autophagy, mainly mitophagy, were noted at all investigated levels of the retinal organization. In particular, in photoreceptor cells, in the ellipsoids of the inner segments of the rods and cones dendrites the volume densities (VV) of autophagosomes was 4.5% and 0.6%, respectively. There were noted mitochondrial swelling (VV = 4%), expansion of rough endoplasmic reticulum (rER) cisterns (VV = 5.9%), and hypertrophy of the Golgi apparatus (8.3%) in rod neurons. The presence of mitochondrial swelling was observed in the axons of the photoreceptors and dendrites of the bipolar neurons. Autophagosomes were noted in the cytoplasm of retinal glial cells—Müller cells.

**Conclusions:** At the terminal stage of glaucoma, structural signs of organelle damage and autophagy were noted in the cells of the studied retinal layers. The greatest structural changes and the degree of development of autophagy, mainly mitophagy, were found in photoreceptor cells. Mitophagy in retinal neurons under pathological conditions can play a dual role: on the one hand, it promotes cell survival by removing damaged organelles, and on the other hand, it can trigger cell death by apoptosis.

**Disclosure:** The authors confirm that they have no conflict of interest.

## 22

### The autophagy contribution into the cells killing by cold atmospheric plasma irradiation

#### Ekaterina Patrakova^1,2^, Mikhail Biryukov^1,2^, Irina Schweigert^3^, Dmitry Zakrevsky^4^, Olga Troitskaya^1^, Pavel Gugin^4^, Elena Milakhina^4,5^, Olga Koval^1,2^

##### ^1^Institute of Chemical Biology and Fundamental Medicine, Siberian Branch of the Russian Academy of Sciences, Novosibirsk, Russia; ^2^Novosibirsk State University, Novosibirsk, Russia; ^3^Khristianovich Institute of Theoretical and Applied Mechanics, Siberian Branch of the Russian Academy of Sciences, Novosibirsk; ^4^Rzhanov Institute of Semiconductor Physics, Siberian Branch of the Russian Academy of Sciences, Novosibirsk, Russia; ^5^Novosibirsk State Technical University, Novosibirsk, Russia; ORCID: 0000-0001-7788-2249

**Introduction:** Atmospheric pressure plasma (CAP) becomes one of the promising strategy for the development of new anticancer approaches. The activity of CAP against cancer cells are connected with reactive oxygen (ROS)- and nitrogen species which are generated by plasma streamers. The excess of oxidized biological molecules is associated with cellular toxic effects, but the precise mechanisms that lead to CAP-induced cell death are largely unknown. In addition to ROS, CAP irradiation is accompanied with UV radiation and electromagnetic fields. Recently, it has been demonstrated that ROS can induce autophagy leading to the death of cancer cells, meaning that autophagy can make a contribution to CAP-induced cell death. In this work we have analyzed autophagy-related hallmarks in CAP-treated human lung cancer cells A549 and normal lung fibroblasts WI-38.

**Materials and methods:** In our experiments, cold atmospheric plasma jet is generated by the plasma device which is a quartz tube with the powered pin electrode inside and grounded ring electrode over the tube next to the nozzle. The inert gas helium is pumping through the plasma device with the rate of 1-10 L/min. The voltage amplitude ranges from 2.6 kV to 5 kV and frequency is 13–40 kHz.

Death of irradiated cells was analyzed by real time iCelligence system and by flow cytometry with AnnexinV/PI staining. Autophagy-related proteins were analyzed by Western blot, H2O2 relative amount was analyzed by flow cytometry using DCFDA, acidic vesicular organelles were detected by flow cytometry.

**Results:** The analysis of autophagy-related hallmarks was made under optimized CAP conditions (duration 60 c, voltage amplitude 4.2 kV, 3 L/min in helium) when normal fibroblasts WI-38 stayed alive and A549 cancer cells were killed. Under indicated conditions, H_2_O_2_ level was significantly increased in irradiated A549 cancer cells. We revealed that CAP irradiation induced the increase of acidic vesicular organelles and lipidation of LC3 in both normal and cancer cells that indicates the autophagy in treated cells. Autophagy inhibitor chloroquine promotes cell death in A549 CAP-irradiated cells, but not in WI-38 cells.

**Conclusions:** Taken together, CAP induces ROS-related cell death in transformed and cancer cells with autophagy markers but failed to induce autophagic cell death in non-transformed cells.

**Funding:** The authors gratefully acknowledge financial support from Russian Science Foundation, grant N 19-19-00255. The authors, EE and DmZ, were partly supported financially by RFBRN18-08-00510 for development of various designs of experimental setup.

**Disclosure:** The authors confirm that they have no conflict of interest. The work was supported by the Russian Science Foundation, grant# 19-19-00255.

## 23

### Attenuation of Set7/9 decreases autophagy in breast cancer cells and promotes their sensitivity to genotoxic therapy

#### Oleg Semenov^1^, Oleg Shuvalov^1^, Matvey Ivanov^1^, Arsenia Zharova^1^, Alexey Petukhov^2^, Alexandra Daks^1^

##### ^1^Institute of Cytology Russian Academy of Science, Saint-Petersburg, Russia; ^2^Almazov National Medical Research Centre, Saint-Petersburg, Russia; ORCID: 0000-0003-0495-1244

**Introduction:** The most common cancer type diagnosed among the female worldwide is the breast cancer (BC). Recently the correlation between high Set7/9 level and decreased lifespan of BC patients was shown. Set7/9 has various targets and regulates such key cellular processes as cell cycle regulation, genotoxic stress response, and apoptosis. The cytotoxic agents like cisplatin and doxorubicin are the common anticancer drugs. Their effect depends on the ability of cancer cells to undergo the apoptosis in response to DNA damage. One of the mechanisms that support cancer cell survival and inhibits apoptosis under genotoxic stress conditions is autophagy. The understating the role of Set7/9 in autophagy-apoptosis crosstalk can help to improve a cytotoxic treatment. Thereby, our purpose was to estimate the role of Set7/9 in regulation of autophagy and apoptosis in breast cancer cells in response to DNA damage.

**Materials and methods:** We obtained the SKBR-3 and MCF-7 BC cell lines with knockdown of Set7/9 using lentiviral transduction with anti-Set7/9 shRNA vector. We used MCF-7 and SKBR-3 because these two cell lines belong to different subtypes: luminal A (LA) MCF-7 cell line is HER2-negative with wild type of p53 expression, while SKBR-3 cells are characterized by mutated p53 and high expression of HER2 receptor, and correspondingly refers to HER2-positive subtype [3]. We investigated the autophagy level in BC cells with different Set7/9 status by WB analysis of LC3 autophagy marker and flow cytometry with lysotracker staining. As a result, we showed that Set7/9 knock-down (Set7/9 KD) caused the decrease of autophagy level in both cell lines. We also revealed the resistance to autophagy inhibitor chloroquine treatment of Set7/9 KD cell lines compared to the control cell lines.

**Results:** Further, we examined the effect of Set7/9 KD on cytotoxic treatment sensitivity. We demonstrated that Set7/9 KD contributed to increased sensitivity to doxorubicin and cisplatin of both investigated cell lines. We additionally performed a flow-cytometry assay with annexin-5 staining to reveal the mechanism of cell death induced by genotoxic drugs. As a result, we showed that Set7/9 induces apoptosis under DNA damage conditions.

Thereby, we determined the role of Set7/9 as positive regulator of autophagy in breast cancer cell. The effect of Set7/9 KD was shown for both LA and HER2-positive BC cells. We speculate that lower level of autophagy in Set7/9-deficient cells Results. in blocking of negative regulation of apoptosis that lead to higher sensitivity to genotoxic drugs. This study contributes to understanding the development of BC resistance to DNA damaging agents and reveals Set7/9 as a potential target for development the BC therapy.

**Funding:** This work was partially supported by Russian Science Foundation grant No 21-75-10138.

**Disclosure:** The authors confirm that they have no conflict of interest. This work was partially supported by Russian Science Foundation grant No 21-75-10138.

## 24

### Epithelial-mesenchymal transition in ZEB1 overexpressing MCF7 cells and regulation of autophagy

#### Julian Rozenberg^1^, Eugene Tulchinsky^1^, Emre Sayan^1^, Alexander Kagansky^1^, Nickolai Barlev^1^

##### ^1^Moscow Institute of Physics and Technology, Dolgoprudny, Russia; ORCID: 0000-0002-7627-5217

**Introduction:** Autophagy is a survival strategy adopted by breast cancer upon treatment. How autophagy influences and is influenced by the epithelial–mesenchymal transition of breast cancer cells is controversial (. We addressed this question by overexpression of ZEB1 in MCF-7 breast cancer cells and by investigating how ZEB1 induced EMT influences autophagy-related gene expression. Tetracycline-induced ZEB1 overexpression promoted EMT and inhibited cell cycle progression at mainly G0/G1 but also G2/M phases. Among others, 75 out of 450 genes related to autophagy were repressed and 25 autophagy genes were upregulated by the ZEB1 induction. For example, Beclin1, a key component promoting autophagy, was 2.4x repressed, however, the level of RNA expression was still high, in the top 50% of expressed genes. Moreover, genes encoding key MTORC1 signaling pathway proteins mTOR, RRAGB. RRAGC, RRAGD, FLCN, and ATPase subunits, all were also repressed upon ZEB1 expression.

**Materials and methods:** This suggests that MTORC1 assembly at the lysosome, amino acid sensing pathways are inhibited, and autophagy can be activated if other pathway components are unchanged, thereby providing a negative feedback loop regulating the level of autophagy. Indeed, LC3 -1 and LC3-2 protein levels were unchanged upon ZEB1 induction.

**Results:** Overall, RNA expression data suggest that expression of key autophagy components and related pathways change upon ZEB1 induced EMT in MCF7 breast cancer cells, however, we did not detect significant changes in the autophagy levels.

**Funding:** The authors gratefully acknowledge financial support from RSF, grant # 20-15-00189.

**Disclosure:** The authors confirm that they have no conflict of interest. The work was supported by RSF, grant # 20-15-00189.

## 25

### Possible role of transglutaminase 2 in chromatin variations under DNA damaging conditions

#### Evgenii Smirnov^1^, Yuliya Gnennaya^1^, Ekaterina Lomert^1^, Nickolai Barlev^1^, Dmitri Tentler^1^

##### ^1^Institute of Cytology of the Russian Academy of Sciences, Saint Petersburg, Russia; ORCID: 0000-0002-6047-5652

**Introduction:** Transglutaminase 2(TG2) is known to be a Ca^2+^-dependent enzyme with multiple activities such as isopeptidase, amine incorporating, crosslinking, protein disulfide isomerase activities. Due to multiple activities, TG2 takes part in a wide range of signaling pathways and is associated with a plethora of diseases including fibroproliferative, neurodegenerative, cardiovascular diseases, and cancer. Additionally, TG2 is involved in mediating chemotherapy resistance and DNA damage repair. Recently, TG2 was shown to post-translationally modify histone H3K9me3 implementing the former in chromatin regulation.

**Materials and methods:** In our research, we studied the role of TG2 in DNA damage response. We performed co-immunoprecipitation of the TG2 protein in hepatocellular carcinoma Huh7 cells treated with doxorubicin. We found that there is an interaction not only between TG2 and y-H2A.X protein, the major marker for DNA damage, but also between TG2, p53, and modified y-H2A.X. The latter is supposedly ubiquitinylated. Additionally, we showed that in doxorubicin-treated mouse embryonic fibroblasts (MEF) TG2 translocated from the nucleus to the cytoplasm, unlike in cancer cell lines where it accumulates in the nucleus after doxorubicin treatment. We also detected multi-directional changes in histone modifications inflicted by loss of TG2. In the knockout cells there is lowered trimethylated histone H3K9 and H3K36, however, trimethylated H3K4 and mono- and di-methylated H4K20 are increased. All of the methylated histones listed above are involved in DNA damage response. Future experiments will be conducted to obtain a better understanding of the role of TG2 in DNA damage response.

**Funding:** The work was supported by the Mega-Grant Program 14.W03.31.0029 and the RSF research project 19-45-02011.

**Disclosure:** The authors confirm that the project was supported by the Mega-Grant Program 14.W03.31.0029 and the RSF research project 19-45-02011.

## 26

### Electron microscopic analysis of autophagy in neurons with expanded CAG repeats in the huntingtin gene in patient-specific and transgenic cell model

#### Lyubov Suldina^1^, Ksenia Morozova^1^, Tuyana Malannova^1^, Anastasia Malakhova^1^, Elena Kiseleva^1^

##### ^1^Institute of Cytology and Genetics, Siberian Branch of the Russian Academy of Sciences, Novosibirsk, Russia; ORCID: 0000-0001-6949-182X

**Introduction:** Huntington’s disease is an inherited neurodegenerative disease caused by an increased number of CAG repeats in the huntingtin (*HTT*) gene; this mutation leads to the formation of protein aggregates and the death of medium spiny neurons in the striatum. Huntingtin is involved in a variety of cellular processes, in particular in the regulation of autophagy. The aim of this work was to investigate and compare the ultrastructural organization of autophagic components between specific spiny neurons prepared by differentiation of induced pluripotent stem cells (IPSC) derived from patient’s mononuclear cells (with 47 CAG repeats) and transgenic neurons prepared by differentiation of IPSc derived from fibroblasts carrying an insertion of 69 CAG repeats that was introduced into the *HTT* gene by the CRISPR/Cas9 method. Neurons without additional insertions into huntingtin served as an isogenic control and were prepared by differentiation of fibroblasts.

**Materials and methods:** The method for obtaining mutant spiny neurons was similar to that described earlier for HEK293 cells. For transmission electron microscopy, cells were grown on plastic films, fixed with 2.5% glutaraldehyde in 0.1 M cacodylate buffer, then incubated with 1% OsO4 in the same buffer, dehydrated, and embedded in Epon. Transmission electron microscopy was carried out at the Multi-Access Center for Microscopy of Biological Objects at the Institute of Cytology and Genetics of the Siberian Branch of the Russian Academy of Sciences using a JEM1400 microscope (Japan).

**Results and conclusions:** Control neurons contained small lysosomes up to 0.2 μm in diameter, a small number of autophagosomes of different sizes, and autolysosomes with inclusions of cytoplasmic organelles as well as light vacuoles. In patient-specific neurons with 47 CAGs and isogenic mutant neurons with 69 CAGs, the number of small lysosomes was not higher. The number of large autolysosomes (>0.6 μm in diameter) with inclusions of organelle fragments as well as light vacuoles sometimes surrounded by smooth endoplasmic-reticulum membranes was 5-fold higher in mutant cells with 69 CAG repeats. Nonetheless, in mutant neurons of both lines, we observed impaired integrity of membranes of large autolysosomes and (sometimes) of vacuoles and a release of their contents into the cytoplasm. In some neurons, accumulation of large autolysosomes with electron-dense contents was detected. The Results. indicate dysfunction of components of the autophagic system at late stages of their formation and accumulation of undigested autophagic structures in neurons. The increased number of CAG repeats caused greater damage to autolysosomal and vacuole membranes, thus possibly resulting in neuronal death. Our findings are consistent with the modern concept of disturbances of late-stage autophagy in neurodegenerative diseases.

**Funding:** This work was supported by ICG funded projects No. 0342-2019-0042 and 0259-2019-0008-C-01 and by the Russian Science Foundation grant (No. 16-15-10128П).

**Disclosure:** The authors confirm that they have no conflict of interest. This work was supported by ICG funded projects No. 0342-2019-0042 and 0259-2019-0008-C-01 and by the Russian Science Foundation grant# 16-15-10128П.

## 27

### Transglutaminase 2 is involved in the DNA repair process

#### Dmitri Tentler^1^, Ekaterina Lomert^1^, Yuliya Gnennaya^1^, Giomar Vasilyeva^1^

##### ^1^Institute of Cytology of the Russian Academy of Sciences, Saint Petersburg, Russia; ORCID: 0000-0003-3976-1205

**Introduction:** Transglutaminase 2 (TG2) is a broadly expressed multifunctional enzyme that possesses a number of activities, including protein disulfide isomerase, protein kinase, G-protein, and some others. Various enzymatic activities and ubiquities expression seem to result in variety of intracellular functions. TG2 involvement in autophagy, apoptosis, mitochondrial functioning, heat shock response has been established. Recent findings suggest its critical role in tumor growth. To understand the oncosupressive mechanisms of TG2 ablation, we investigated DNA repair process in mouse embryonic fibroblasts obtained from the knock-out mice (MEF TG2 KO) and the wild-type controls (MEF WT).

**Materials and methods:** To introduce DNA breaks, we treated cells with genotoxic anti-cancer agents, doxorubicin, and etoposide. Both agents interfere with topoisomerase II but have slightly different effect on the double/single-strand breaks ratio. The cells were treated for a short time (40 min). We then removed the genotoxic agents, and monitored the DNA repair process. Additionally, X-Ray and UV irradiation was applied as non-chemical ways to introduce DNA breaks.

At first, we studied phosphorylation of H2AX histone (γ-H2AX) using high throughput microscopy. Intensity of γ-H2AX staining, mean number of foci per nucleus, mean focus area and intensity were estimated. We found that γ-H2AX staining and number of γ-H2AX foci were lower in MEF TG2 KO cells regardless of the genotoxic agent. The differences between MEF WT and TG2 KO cells were more profound during the first hours after treatment, and almost flattened after 12 h. Since γ-H2AX is just a marker of the DNA breaks, we have tested whether the repair complexes are also affected. Immunostaining for 53BP1 protein showed that the number of the

53BP1 foci in MEF WT was constantly decreasing from 2 to 12 hours after treatments, whereas TG2 KO cells showed significant delay in the foci formation. Thus, we found that TG2 deficiency affects both H2AX phosphorylation and 53BP1 foci formation upon genotoxic stress.

Our second task was to investigate whether differences between TG2 KO and WT MEFs in immunostaining for DNA repair proteins do correspond to impairments in the DNA repair process. We monitored changes in the DNA fragmentation after genotoxic stress using the comet assay approach. This way, we directly estimated recovery of the genomic DNA integrity over time. The investigation showed that DNA fragmentation is evenly high in WT and TG2 KO MEFs just after the doxorubicin or etoposide treatment. These data suggest that there is no difference in the very process of the DNA breaks formation. However, TG2 KO cells display higher fragmentation after 8 h recovery, which further supports TG2 involvement in the DNA repair.

**Results and conclusions:** Finally, we investigated whether re-introduction of TG2 into the TG2 KO MEFs may increase the DNA repair. The γ-H2AX staining was estimated in TG2 KO and KO/KI (knock- out/knock-in) MEFs after the doxorubicin and etoposide treatments. The Results. showed that both signal intensity and number of foci are higher in KO/KI cells, which further supports direct TG2 involvement in the DNA repair process. The on-going studies should reveal TG2 interactions with the DNA repair complexes, and develop new TG2-based approaches to increase cancer cells sensitivity to the genotoxic therapy.

**Funding:** This work was supported by the grant from the Russian Government Program 14.W03.31.0029.

**Disclosure:** The authors confirm that the project was supported by the Grant from Russian Government (Mega Grant) 14.W03.31.0029.

## 28

### Gibberellic acid induces autophagy and differentiation in human epidermoid carcinoma cell line A431 via activation of endoplasmic reticulum stress

#### Mariya Vildanova^1^, Polina Vishnyakova^2,3^, Ekaterina Turishcheva^1^, Alina Saidova^1^, Victoria Konduktorova^1^, Daria Potashnikova^1^, Galina Onishchenko^1^, Elena Smirnova^1^

##### ^1^Lomonosov Moscow State University, Faculty of Biology, Russia; ^2^National Medical Research Center for Obstetrics, Gynecology and Perinatology Named after Academician V.I. Kulakov of Ministry of Healthcare of Russian Federation; ^3^Peoples’ Friendship University of Russia; ORCID: 0000-0002-8980-6988

**Introduction:** Plant hormones and their natural and synthetic derivatives are promising objects of investigation, because they impact viability and metabolism of human cells. Plant hormone gibberellic acid (gibberellin A3) (GA), potent plant growth regulator, is commonly used in agriculture. It turned out that GA can have different effects on the cells of animals and humans, depending on the object of study, concentrations, and application regimen. Many studies report the toxic, carcinogenic, and allergic effects of GA, but emerging evidence indicates that GA and its derivatives may cause anti-inflammatory and anti-tumor activity, stimulation of secretion.

**Materials and methods:** This work was aimed to investigate the possibility of ER stress activation by GA and to reveal the differences in the responses to GA of human cultured immortalized non-tumorigenic HaCaT and epidermoid carcinoma A431 cells. We researched the effect of GA (2 mM, 24 h) on both cell lines. Using flow cytometry and Annexin V/propidium iodide labeling, we found that GA does not affect cellular viability and proliferation. RT-qPCR showed that GA enhances/elevates the expression of genes associated with ER stress—*GRP78* and *CHOP* in HaCaT cells, and *GRP78*, *ATF4*, and *CHOP* in A431 cells. Intracellular Ca^2+^ concentration was assayed by the Fluo-4 AM calcium probe. The addition of GA was accompanied by the influx of intracellular Ca^2+^ in both cell lines. However, Western blot analysis showed that the content of ER stress marker GRP78 increased only in A431 cells. Mild ER stress can activate autophagy and cellular differentiation. The increased content of autophagy marker LC3B-II was revealed only in GA-treated A431 cells. Using transmission electron microscopy, we showed the different phases of autophagic flux (autophagosomes and autolysosomes) in control and GA-treated A431 cells.

**Results and conclusions:** It demonstrates the progression of autophagic flux without arrest in the early stages. Immunocytochemical staining and Western blot analysis demonstrated that GA selectively increased the level of keratinocytes differentiation markers: involucrin—only in A431 cells, and filaggrin—in both cell lines. Additionally, normalization of involucrin staining was observed in A431 cells. Thus, we demonstrated for the first time that plant hormone GA induced mild ER stress with different outcomes for normal and tumor cells: adaptive response is manifested in both HaCaT and A431 cells, but autophagy and differentiation—only in A431 cells.

**Disclosure:** The authors confirm that they have no conflict of interest.

## 29

### Preferential accumulation of EGFR-specific nanoMIPs in autophagosomes of pancreatic cancer cells

#### Konstantin Shevchenko^1,2^ and Larissa Lezina^3^

##### ^1^Institute of Biomedical Chemistry, Moscow, Russia; ^2^Institute of Cytology RAS, St. Petersburg, Russia; ^3^Moscow Institute of Physics and Technology, Dolgoprudny, Moscow region, Russia; ORCID ID: 0000-0003-2411-7636

**Introduction:** Autophagy is the molecular mechanism of intracellular destruction which is activated in response to ER- and mitochondrial-induced stress. Autophagy is believed to play both tumorigenic and tumor suppressive functions. Recent developments in nanomedicine introduced novel therapeutic approaches based on the use of nanoparticles. Molecularly imprinted polymers (MIPs) are acryl-based polymeric nanoparticles that carry imprints of the template molecule and hence can specifically bind the corresponding peptide targets on the cell surface. This feature has made them one of the key candidates for substitution of the antibodies in diagnosis and therapy of various diseases. Compared with antibodies MIPs have better cost-effectiveness, higher stability and easier in synthesis. We have recently shown that MIPs loaded with doxorubicin against EGFR were effective in specific killing those cancer cells that express this receptor. Importantly, one of the biomarkers of pancreatic ductal adenocarcinoma (PDAC), which is one of the most aggressive and therapy-resistant tumor types, is overexpression of EGFR. Despite our initial evidence that EGFR-specific MIPs induce cell death, there is no understanding how MIPs exert this effect. To address this question, we have studied the intracelullar uptake of MIPs by cancer cells using confocal microscopy.

**Materials and methods:** The MIPs were developed against 16-mere oligopeptide of the EGFR protein. The sequence has been chosen based on the analysis of the potential immunogenic sites within the protein sequence. Next, the MIPs were synthesized by copolymerization of the fluorescently labeled acrylamide monomers with methacrylic acid as a cross-linker. The obtained MIPs were characterized using scanning electron microscopy and dynamic light scattering. The binding constant was evaluated using BiaCore assay. Immune fluorescence microscopy was performed using the Zeiss AxioObserver A1 confocal microscope.

**Results and Conclusions:** The obtained spheric cross-linked fluorescent nanoparticles had the mean hydrodynamic size 100 ± 5 nm which was confirmed by SEM. They had excellent thermal stability and survived the autoclave treatment (121 °C, 15 mins). The particles recognized and specifically bound the EGFR peptide at Kd of 5nM as analyzed by BiaCore assay. EGFR-overexpressing cells (PANC-1, MDA-MB-468) showed an increased accumulation of fluorescent MIPs inside the cells as judged by confocal microscopy. Importantly, co-staining of EGFR-MIPs with LC3-specific antibody showed their co-localization in the autophagosomal vesicles. These results suggest that one of the mechanisms how EGFR-MIPs can induce cell death is through induction of autophagy, which potentially can be caused by the ER stress.

**Funding:** The work was done in the framework of the Russian Federation fundamental research program for the long-term period for 2021–2030 to the Institute of Biomedical Chemistry.

**Disclosure:** The MIP technology is proprietary for the MIP Diagnostics Ltd., Sharnbrook, UK.

